# Structure and physicochemical properties of starches in lotus (*Nelumbo nucifera* Gaertn.) rhizome

**DOI:** 10.1002/fsn3.37

**Published:** 2013-05-05

**Authors:** Huaguang Yu, Libao Cheng, Jingjing Yin, Shunjun Yan, Kejun Liu, Fengmin Zhang, Bin Xu, Liangjun Li

**Affiliations:** 1 College of Physics Science and Technology, Yangzhou UniversityYangzhou, 225002, P. R. China; 2 School of Horticulture and Plant Protection, Yangzhou UniversityYangzhou, 225009, P. R. China; 3 Testing Center, Yangzhou UniversityYangzhou, 225009, P. R. China

**Keywords:** Crystalline structure, lotus (*Nelumbo nucifera* Gaertn.) rhizome, solid-state nuclear magnetic resonance, starch, X-ray powder diffraction

## Abstract

The type and content of starch are believed to be the most critical factors in determining the storage and processing quality of lotus rhizome species, and the intention of this study is to survey the structure and properties of starches isolated from rhizomes of two lotus cultivars using X-ray powder diffraction, solid-state nuclear magnetic resonance spectroscopy, attenuated total reflectance-Fourier transform infrared spectroscopy, scanning electron microscope, differential scanning calorimetry, and rapid viscosity analyzer (RVA). Starch in rhizome of cultivar Meirenhong exhibited C-type X-ray diffraction pattern, while starch in rhizome of cultivar Wawalian showed A-type pattern. ^13^C cross-polarization magic-angle spinning nuclear magnetic resonance (^13^C CP-MAS NMR) also confirmed the polymorphs. The relative crystallinity of two starches was quantitatively estimated from two methods and compared. Attenuated total reflectance-Fourier transform infrared (ATR-FTIR) results indicated that the external regions of the starch granules had a great level of ordered structure. Starch granules in Meirenhong showed oval-shaped granules, while starch granules in Wawalian were elongated and oval in shape with relatively large size. Gelatinization temperatures of starch in Meirenhong and Wawalian were 330.5 and 342.4 K, respectively, and the gelatinization temperature range of Meirenhong was significantly wider than that of Wawalian. Starch in rhizome of cultivar Meirenhong showed lower pasting temperature, lower hot and cool viscosities, lower setback, and higher peak viscosity and breakdown than those of Wawalian in RVA pasting profiles at 6% starch concentration.

## Introduction

Lotus (*Nelumbo nucifera* Gaertn.), a member of the family Nymphaeaceae, is an aquatic herb vegetable. Lotus has been wildly cultivated in China, Japan, and other Southeast Asian countries for its multiple purposes. Lotus rhizome contains abundant of nutrients including starch, protein, amino acids, dietary fiber, vitamins, and mineral substances, and is widely favored by Asian people as functional foods (Chiang and Luo 2007). The products of lotus rhizome such as fresh, salted, and boiled lotus rhizome, lotus rhizome starch, drinks, teas, and lotus seeds are very popular in the daily diet (Hu and Skibsted 2007; Zhong et al. 2007).

With the unique characteristics, lotus rhizome forms underground. Lotus rhizome plant develops several rhizomes in a single growing season with average length of 10–20 cm each. Large quantities of nutrients are produced and stored in these rhizomes (Masuda et al. 2006, 2007). In the previous study, we investigated the rhizome formation in the physical and biochemical context in detail (Li et al. 2006). Starch is the most important component found in the storage organ rhizome with average content of 10–20% in total fresh weight of lotus rhizome, and varies in different cultivars. The content and variety of starch are believed to be the most critical factors in determining the storage and processing quality of the cultivated species.

Although many varieties are being cultivated, lotus rhizome can be divided into two categories according to its quality. The main features of the first category include its crispiness due to high water content, low starch, high sugar, and low crude fiber content. These characteristics often result in precipitation of starch, gelatinization, and low viscosity during product processing, which leads to crispiness and refreshing taste of good sensory quality. The second type of the lotus rhizome is characterized by high starch, and low water content. High content of starch makes its texture soft, gives it a ductile slip, and increases the viscosity of the products (Wattebled et al. 2002).

Starch is a kind of natural polymer, and starch occurs in nature as semicrystalline granules (Buleon et al. 1998; Liu and Shi 2006; Tang et al. 2006; Corre et al. 2010; Perez and Bertoft 2010). Starch granules occur in all shapes and sizes (spheres, ellipsoids, polygon, platelets, and irregular tubules). They have diameters ranging from around 0.1 to 200 μm depending on their botanical origin (Perez and Bertoft 2010). Under an electron microscope, starch granules show 120- to 400-nm-thick-growth rings of alternating partially crystalline and amorphous material (Buleon et al. 1998; Corre et al. 2010; Perez and Bertoft 2010). The whole starch granule consists of stacks of semicrystalline regions that are separated by amorphous growth rings (Cameron and Donald 1992; Jenkins et al. 1993; Waigh et al. 1997). In each partially crystalline ring, there are alternating crystalline lamellae and amorphous lamellae. The crystalline lamellae comprised double helices formed from outer chains of amylopectin, whereas the amorphous lamellae are made up of glucose units near branch points of the amylopectin molecules (Liu and Shi 2006). The amorphous growth rings between the semicrystalline growth rings are filled with amylose and in some case, amylose-lipid complexes (Tang and Hills 2003). The widely accepted cluster model proposed that amylopectin was composed of alternating regions of amorphous lamellae containing the branch points, and highly crystalline lamellae composed of double helices of outside chains of amylopectin (Buleon et al. 1998). In most common types of starch, the weight percentages of amylose range between 18% and 28%, and amylopectin from 72% to 82% (Corre et al. 2010).

Starches extracted from lotus rhizomes are commercially available in China and consumed as breakfast, fast food, traditional confectionery, and food additives (Zhong et al. 2007). Starches from different botanical sources have diverse physicochemical and functional properties, and are greatly affected by environmental conditions. At present, most studies have been focused on corn, rice, wheat, potato, and tapioca starches. The purpose of this study was to investigate the structure, morphological, thermal, and pasting properties of starches extracted from lotus rhizomes planted in the southeast area of China.

## Materials and Methods

### Plant materials

Two cultivars of lotus, Meirenhong and Wawalian, were obtained from the experimental base of aquatic vegetables of Yangzhou University, Yangzhou, Jiangsu Province, P. R. China. Meirenhong was a lotus species with low starch content, while Wawalian contained high starch in rhizome.

### Preparation of starches

Native starch granules were isolated following the method described in literature (Man et al. 2012). All rhizomes were washed, brushed, and peeled immediately. After peeling, the rhizomes were cut into small pieces and homogenized with water in a household blender. The slurry was filtered through gauze to amass the residue while collected the filtrate in a 500-mL glass beaker. Washed residue left on the gauze with distilled water three times to facilitate the release of starch granules from the fibers, and then discarded the residue. The combined extract was filtered with 100-, 200-, and 300-mesh sieves, respectively. The beakers containing filtrate were kept undisturbed to settle the starch naturally. The supernatant liquid was decanted, and then the sedimentation was transferred into a 50-mL tube and centrifuged at 1500 rpm for 10 min. The yellow gel-like layer on top of the packed white starch granule pellet was carefully scraped off and discarded. The process of centrifugation separation was repeated several times until no dirty material existed. Finally, starches were transferred to clean filter paper, and air dried.

Starch samples were placed in a hermetical desiccator for >15 days before the X-ray powder diffraction (XRD) and nuclear magnetic resonance (NMR) experiments. The desiccator contained a saturated solution of NaCl at 298 K, providing an environment with a relative humidity (RH) of 75%. The H_2_O content was estimated to be about 20 w/w%.

### XRD analysis

XRD measurements of two different lotus rhizome starches were performed in a Bruker AXS D8 ADVANCE X-ray powder diffractometer operating at 40 kV and 30 mA at ambient temperature. Cu K*α*_1_ radiation (*λ* = 0.15405 nm) was used. The scanning region of the diffraction angle (2*θ*) was from 3° to 35° with a step size of 0.02°, and a scan rate of 0.5° min^−1^. The empty sample holder was scanned under the same conditions as the samples so that the instrumental background could be determined. The relative crystallinity of samples was quantitatively estimated following the method of Cairns, Bogracheva, Ring, Hedley, and Morris (Cairns et al. 1997).

### Solid-state NMR analysis

All the solid-state NMR experiments were carried out at *B*_0_ = 9.4 T on a Bruker AVANCE III 400 WB spectrometer. The corresponding resonance frequency of ^13^C was 100.6 MHz. Samples were packed in a 7-mm ZrO_2_ rotor and spun at the magic angle (54.7^o^), and the spin rate was 6 kHz. ^1^H-^13^C CP-MAS spectra were acquired with a contact time of 1.2 msec and a recycle delay of 2 sec. The ^13^C chemical shift was externally referenced to the high field resonance of hexamethylbenzene at 17.17 ppm. The deconvolution of the NMR spectra was conducted using the program PeakFit™ version 4.12 (Systat Software Inc., CA). The relative crystallinity of samples was calculated according to the method described by Paris, Bizot, Emery, Buzare, and Buleon (Paris et al. 1999).

### ATR-FTIR measurement

Attenuated total reflectance-Fourier transform infrared (ATR-FTIR) spectra were recorded in a Varian Cary 670 FTIR spectrometer with a deuterated triglycine sulfate detector equipped with an attenuated total reflectance single reflectance cell with a germanium crystal (45° incidence-angle) (PIKE Technologies). Starches were dispersed in water (70% water w.b.) prior to FTIR analysis. Samples were measured directly after pressing the samples on the crystal. For each measurement, 32 scans with a 1 cm^−1^ resolution were adopted before Fourier transformation. The spectrum of water recorded in the same conditions was subtracted from the sample spectra. The Lorentzian line shape with a half-width value of 19 cm^−1^ and a resolution enhancement of 2.0 was used in deconvolution. Infrared (IR) absorbance values at 1047, 1022, and 995 cm^−1^ were extracted from the spectra after water subtraction, baseline correction, and deconvolution. Intensity measurements were performed on the deconvoluted spectra by recording the height of the absorbance bands from the baseline. For the purpose of comparison of IR spectra, the vector normalization function was used in the region 1075–950 cm^−1^.

### Morphology observation of starch granules

Morphology of lotus rhizome starch granule was obtained on a Hitachi S-4800 scanning electron microscope (SEM) with an acceleration voltage of 15 kV. Starch samples were suspended in anhydrous ethanol to obtain a 1% (w/v) suspension. One drop of the suspension was mounted on a circular aluminum stub with double-faced adhesive tape, and then coated with gold. A large amount of images were obtained from different areas to observe the morphology of starch granules. Starch granule diameter measurements were taken using SEM image scale bars.

### DSC analysis

Before differential scanning calorimetry (DSC) experiments, the starch samples were placed in an electrically heated drying cabinet with constant temperature (about 343 K) for about a week. Dry starch (about 4 mg) was weighed accurately in aluminum crucibles and 12 μL deionized water was then mixed in (i.e., maximum 25%, w/w of starch). The mixture was hermetically sealed in aluminum crucibles and kept in an icebox at 273 K overnight. After equilibrating at room temperature for 1 h, the crucibles were heated from 293 to 413 K at a rate of 3 K min^−1^ in a Netzsch DSC 200 F3 differential scanning calorimeter, and an empty crucible was used as a reference. Onset (*T*_o_), peak (*T*_p_), and conclusion (*T*_c_) temperature, and the enthalpy change (Δ*H*) of starch gelatinization were calculated using the software provided.

### RVA analysis

The viscoamylographs of the starches were determined using a Rapid Viscosity Analyzer (RVA 3D, Newport Scientific, Australia). 1.5 g of each sample was weighted, and then put into a new canister which contained 25.0 g of deionized water to prepare a 6% suspension on a dry weight basis (w/w). A programmed heating and cooling cycle was used. The idle temperature was set at 323 K, and the following test profile was run: (1) held at 323 K for 1.0 min, (2) heated to 368 K at a rate of 6.0 K min^−1^, (3) held at 368 K for 5.0 min, (4) cooled to 323 K at a rate of 6.0 K min^−1^, and (5) held at 323 K for 2.0 min. The samples were equilibrated by rotating the paddle at a speed of 960 rpm for the first 10 sec, and then rotating at a constant speed of 160 rpm in the test. Peak viscosity, hot viscosity, breakdown, final viscosity, setback, peak time, and pasting temperature were recorded. All the viscosity parameters were expressed in centipoise (cP).

## Results and Discussion

### XRD patterns of starches isolated from different lotus rhizome

X-ray diffraction is one of the most effective methods in studying the structure of native starch, especially in determining the crystalline form of starch (Cheetham and Tao 1998; Blazek and Gilberta 2011). X-ray diffraction provides an elucidation of the long-range molecular order, typically termed as crystalline, which is due to ordered arrays of double helices formed by the amylopectin side chains (Buleon et al. 1998; Perez and Bertoft 2010). Two different polymorphic forms are commonly observed in native starches, namely, A-type and B-type polymorphs, which consist of parallel-packed, left-handed double helices. In the A-type structure, left-handed parallel-stranded double helices are packed in the monoclinic space group B2. In the B-type structure, however, the double helices are packed in a hexagonal unit cell with the P6_1_ space group (Buleon et al. 1998; Perez and Bertoft 2010). The main difference between A- and B-type is that the former adopts a close-packed arrangement with water molecules between each double-helical structure, while the B-type is more open, there being more water molecules, essentially all of which are located in a central cavity surrounded by six double helices (Buleon et al. 1998; Perez and Bertoft 2010). Gerard et al. (2000) confirmed that the distance between two linkages and the branching density inside each cluster are determining factors for the development of crystallinity in starch granules. Clusters with numerous short chains and short linkage distance produce densely packed structure, the A allomorphic type. Longer chains and distances lead to a B-type. C-type starch pattern has been considered a mixture of both A- and B-types because its X-ray diffraction pattern can be resolved as a combination of the previous two. A third form reported for starch molecules is V-type polymorph, which is a single, left-handed helix often with a complexing agent included in the helical channel (Buleon et al. 1998; Perez and Bertoft 2010). In general, V-type conformation is more easily discovered in high amylose starches. Each form of crystalline can be unambiguously identified from characteristic X-ray diffraction patterns and by ^13^C cross-polarization magic-angle spinning nuclear magnetic resonance (CP-MAS NMR) spectroscopy (Buleon et al. 1998; Perez and Bertoft 2010). X-ray diffraction pattern of starch granule would be mostly affected by botanical source. In the native granular forms, A pattern is mainly associated with cereal starches, B form is usually obtained from tuber starches, and C pattern is related to smooth pea starch and various bean starches (Buleon et al. 1998; Perez and Bertoft 2010).

**Figure 1:**
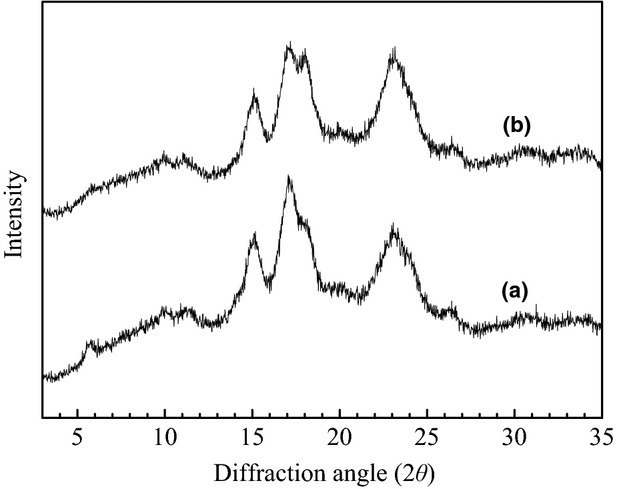
X-ray diffraction patterns of (a) Meirenhong and (b) Wawalian.

From the XRD patterns of two samples (Fig. 1), obvious distinction could be found. A small peak around 2*θ* about 6.3° belonged to B-type crystalline was found for Meirenhong sample. Besides, additional A-type peaks were also observed. At 2*θ* about 23°, only one peak appeared and the peak at about 18° was a shoulder. Therefore, the diffraction peak positions found in the pattern from Meirenhong sample closely approximated to a combination of A- and B-type patterns. Thus, the starch isolated from Meirenhong lotus rhizome was classed as C-type. The starch from Wawalian lotus rhizome showed a typical A-type pattern, with strong reflection at 2*θ* about 15, 17, 18, and 23°.

Both B-type and C-type crystalline have been reported for lotus rhizome starch. For example, Suzuki et al. (1992) and Zhong et al. (2007) reported that lotus rhizome starches showed B-type crystalline, while (Sung et al. 1978; Lii and Lee 1993; Lin et al. 2006; Man et al. 2012) suggested that the lotus rhizome starch had C-type crystalline. These differences suggested that the crystalline structure of lotus rhizome starch was easily affected by temperature and some other conditions (Zhong et al. 2007). In this work, C- and A-type crystalline were observed for the two samples. To the best of our knowledge, this is the first time that A-type crystalline was observed in the starch isolated from lotus rhizome. Man et al. (2012) obtained starches from lotus seed with A-type pattern.

Crystallinity, which can be interpreted as long-range order, in a semicrystalline biopolymer like starch, is defined as the ratio between the mass of the crystalline domains and the total mass of the material. The semicrystalline diffraction pattern is composed of crystalline diffraction peaks superimposed on an amorphous background (Cairns et al. 1997). Several investigators have used indices of relative crystallinity based on methods, in which the area of the crystalline diffraction relative to the total area of the diffractogram is taken as a measure of crystallinity (Cairns et al. 1997; Cheetham and Tao 1998; Blazek and Gilberta 2011). Previous experiments indicated that the degree of crystalline in native starch granule was normally about 15–45% (Buleon et al. 1998). The relative crystallinity of the samples was quantitatively estimated following the method of Cairns (Cairns et al. 1997). The diffraction patterns from the crystalline portions of the starches were obtained after removal of instrumental and amorphous backgrounds. The results will be discussed with the results of the relative crystallinity of the starches determined by ^13^C CP-MAS NMR experiments.

### ^13^C CP-MAS NMR Spectra

Solid-state NMR spectroscopy was widely used in analysis of composition, conformation, crystalline, and gelatinization of starches (Gidley and Bociek 1985; Bogracheva et al. 1998, 2001; Tan et al. 2007). As introduced in the previous research, there are four typical signal areas such as C1, C4 (amorphous), C2, 3, 4, 5, and C6 region in ^13^C NMR spectra of starch (Paris et al. 1999; Tang and Hills 2003). Each of the part represents unique carbon atoms in glucose. Generally, we should pay attention to the C1 region, not only there are no overlapping signals from other carbon but also the glycosidic torsion angle is relatively more straightforward. In previous reports, a characteristic triplet in C1 area attribute to A-type crystalline, which adopts a twofold packing symmetry and leads to three inequivalent residues per unit. While the typical B-type crystalline polymorph composition will show a characteristic doublet in C1 region of ^13^C NMR spectra, just because the threefold symmetry of adjacent helices which belongs to B-type leads to two different residues per unit (Buleon et al. 1998). Since C-type starches have both A- and B-type crystallites, it can be suggested that the peak positions of their ordered parts should be the sum of those for the ordered parts of A- and B-type starches (Bogracheva et al. 1998; Buleon et al. 1998; Perez and Bertoft 2010).

**Figure 2:**
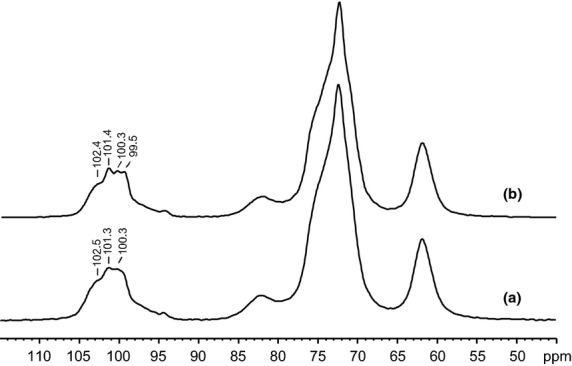
^13^C cross-polarization magic-angle spinning nuclear magnetic resonance (^13^C CP MAS NMR) spectra for (a) Meirenhong and (b) Wawalian.

^13^C CP-MAS NMR spectra of the starches (Fig. 2) could be represented as the sum of the patterns obtained from amorphous and crystalline structure of the starches. The broad resonances at 102.5 and 97.8 ppm (C1), and 82.0 ppm (C4) assigned to amorphous part of native starch were clearly present on both samples (Paris et al. 1999). The differences at C1 area (chemical shift from 105 to 94 ppm) could be discovered in ^13^C CP-MAS NMR spectra of the two samples. We could find two peaks in C1 area of Meirenhong sample at 100.3 and 101.3 ppm, while typical triple peaks at 99.5, 100.3, and 101.4 ppm were observed for Wawalian sample. But when we paid attention to C1 area, Meirenhong sample had an unresolved shoulder at about 99.5 ppm. Therefore, we considered that starches isolated from Meirenhong and Wawalian lotus rhizome were C-type and A-type crystalline, respectively. The small peak appeared at 94.4 ppm in both samples was not much broader than the signals for crystalline materials. This signal was displayed in most of ^13^C CP-MAS NMR spectra reported in the literature with little discussion until it was tentatively attributed to constrained conformations. The overlapping signals around 78–68 ppm were associated with C2, C3, C4, and C5, and the resonance at 61.9 ppm was attributed to C6 (Paris et al. 1999; Tang and Hills 2003). The chemical shifts and assignments were given in Table 1.

**Table 1:** ^13^C CP MAS NMR chemical shifts and assignments

Cultivar name	Chemical shifts (ppm)	Assignments
Meirenhong	102.5	C1, amorphous
	101.3, 100.3, 99.5	C1, crystalline
	94.4	C1, constrained conformation
	97.9	C1, amorphous
	82.1	C4, amorphous
	71–76	C2, 3, 4, 5
	61.9	C6
Wawalian	102.4	C1, amorphous
	101.4, 100.3, 99.5	C1, crystalline
	97.7	C1, amorphous
	94.4	C1, constrained conformation
	82.0	C4, amorphous
	71–76	C2, 3, 4, 5
	61.9	C6

^13^C CP-MAS NMR, ^13^C cross-polarization magic-angle spinning nuclear magnetic resonance.

^13^C NMR spectra of native starches are typically analyzed as a composite of spectra from the amorphous (single chain) and the ordered double-helical components (Gidley and Bociek 1985). ^13^C NMR spectrum of amorphous starch is often much broader than crystalline starch owing to broader conformational distributions. ^13^C NMR spectrum of amorphous starch is not related to the type of native starch from which it is produced (Bogracheva et al. 2001). Accordingly, the relative proportions of amorphous and ordered double-helical components in starch can be estimated by simulating the spectra of native starch as linear combinations of the subspectra of amorphous and appropriate crystalline polymorph (Gidley and Bociek 1985). Bogracheva et al. (2001) proposed a quantitative method to analyze the content of double-helical conformation in native starch, which is so-called C4-PPA method. It can be seen that the resonance for the C4 site of amorphous phase can be identified more easily than for the other sites. This is mainly because it only slightly overlaps the other peaks, and can, therefore, be fitted relatively accurately. In this approach, the proportion of the peak area for C4 resonance relative to the total area of the spectrum (abbreviated as C4-PPA) of native starch is divided by that of a standard amorphous starch. The result is expressed as a percentage to indicate the relative amount of amorphous material in native starch granules (Bogracheva et al. 2001). But the methods mentioned above ignored the existence of V-type conformation, especially in high amylose starch, which may contain a substantial amount of V-type polymorph. The variation in spectral characteristics for both laboratory prepared amorphous samples and amorphous phase of natural starch granules required further examination. An improved method of analyzing ^13^C NMR spectra of native starches to estimate the relative proportions and nature of amorphous, single V-type, and double-helical components within granules and other starch samples was proposed (Tan et al. 2007). Relative proportions of amorphous, single, and double-helical conformations were estimated by apportioning intensity of C1 peak areas between conformational types on the basis of ordered and amorphous subspectra of the native starch (Tan et al. 2007).

**Figure 3:**
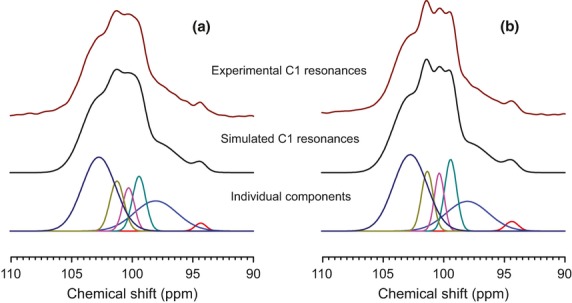
Spectral decomposition of the C1 area (a) Meirenhong and (b) Wawalian.

The percentage of relative crystallinity can be calculated as the proportion of the fitting peak areas of crystalline phase relative to the total area of C1 region (Paris et al. 1999). According to the method, the decomposition of the spectra at C1 area for the two native starches into their respective amorphous and ordered components was conducted using PeakFit software. The spectra at C1 area were decomposed into six resonances and were depicted in Figure 3. The relative crystallinity (%) calculated according to the method and the results estimated from X-ray diffraction method were listed in Table 2 for comparison. The relative crystallinity obtained from ^13^C CP-MAS NMR was slightly higher than those obtained from X-ray diffraction method, which was possibly due to the chain regularity being affected differently by the two techniques (Paris et al. 1999).

**Table 2:** The relative crystallinity determined by XRD and ^13^C CP MAS NMR

Cultivar name	XRD	^13^C CP-MAS NMR
Meirenhong	35 ± 2%	39 ± 2%
Wawalian	37 ± 2%	41 ± 2%

XRD, X-ray powder diffraction; ^13^C CP-MAS NMR, ^13^C cross-polarization magic-angle spinning nuclear magnetic resonance.

### ATR-FTIR spectra

ATR-FTIR is a surface analytical method that can acquire information on the outer region of a sample. The structural properties of surface of starch granule (crystallinity, absorbed non-starch materials, porosity) are suggested to be responsible for the variation in starch granules susceptibility to amylase hydrolysis. IR beam can penetrate into the first few micrometers (about 2 μm) of starch granules. This penetration depth is normally smaller than the average size of starch granules. This implies that the IR spectra acquired are representative of the external part of the starch granules. The alternating growth rings of semi-crystalline and amorphous material are generally around 0.1 mm thick. This means that ATR-FTIR, acquiring on a micron scale, measures the overall information from several growth rings (van Soest et al. 1995; Sevenou et al. 2002). IR is claimed to be sensitive to short-range order, supposed to be the double helix content in starch. X-ray diffraction provides statement about long-range order such as the packing of double helices into ordered arrays. On the other hand, correlations between IR and X-ray diffraction are obtained for mixtures of amorphous and crystalline starch (van Soest et al. 1995; Sevenou et al. 2002). The IR spectrum of starch has been shown to be sensitive to changes in structure on a molecular level (short-range order), such as starch chain conformation, helicity, crystallinity, and retrogradation processes, as well as water content. The IR absorbance band at 1047 cm^−1^ is sensitive to the amount of ordered or crystalline starch, and the band at 1022 cm^−1^ is characteristic of amorphous starch. The ratios of heights of bands at 1047 and 1022 cm^−1^ express the amount of ordered starch to amorphous starch. The IR spectrum and thus the short-range order are also sensitive to water content. In particular, the band at 994 cm^−1^, which is related to intramolecular hydrogen bonding of the hydroxyl group at C6, is water sensitive (van Soest et al. 1995; Sevenou et al. 2002).

**Figure 4:**
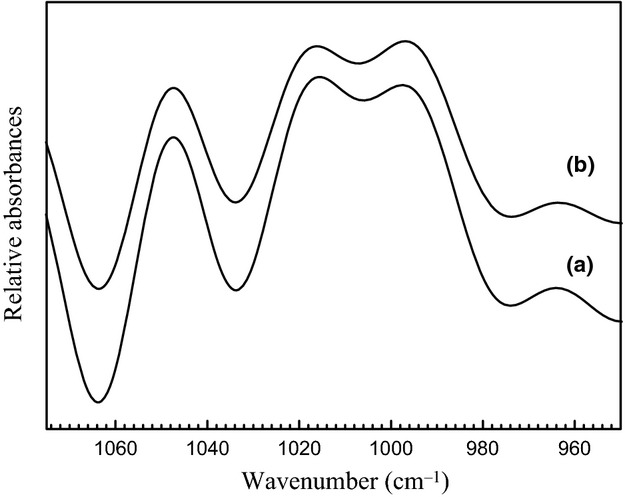
Attenuated total reflectance-Fourier transform infrared (ATR-FTIR) spectra for (a) Meirenhong and (b) Wawalian.

ATR-FTIR spectra in the region 1075–950 cm^−1^ for two starches were shown in Figure 4. It should be noted that the IR spectra for the two samples were very similar. This region of the IR spectrum of starch samples was described by three main modes with maximum absorbance at 1047, 1022, and 995 cm^−1^. The absorbance at the three wavenumbers was obtained from the IR spectra and the ratio of absorbance 1047/1022 and 1022/995 cm^−1^ were calculated and listed in Table 3.

**Table 3:** IR ratio of absorbance 1047/1020 and 1022/995 cm^−1^

Cultivar name	Intensity ratio 1047/1022 (cm^−1^)	Intensity ratio 1022/995 (cm^−1^)
Meirenhong	0.93	0.83
Wawalian	0.92	0.80

IR, infrared.

The high values for the ratio of absorbance 1047/1022 cm^−1^ of the two samples indicated a great level of ordered structure in their external region, which was very similar to amylomaize starch, but the ratio of absorbance 1022/995 cm^−1^ was much larger than that of amylomaize starch (van Soest et al. 1995). From DSC, X-ray diffraction and polarized light microscopy studies of pea starch (C-type) gelatinization in 0.6 mol/L KCl solutions, it could be proposed that B polymorphs were sited in the center of the granules and A polymorphs were located in the outside part of the granules (Bogracheva et al. 1998). In this study, ATR-FTIR spectra acquired were representative of the external part of the starch granules, which was mainly from A polymorphs for Meirenhong sample (C-type). Therefore, the ATR-FTIR spectra for the two samples were very similar.

### SEM micrographs of starch granules

**Figure 5:**
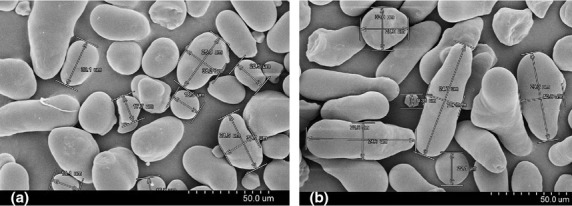
Scanning electron microscope (SEM) images of starch granules (a) Meirenhong and (b) Wawalian.

SEM micrographs of starch granules were presented in Figure 5. Most of starch granules of Meirenhong sample showed oval- and round-shaped granules, while starch granules of Wawalian sample were elongated and oval in shape with relatively large size. Some small or irregularly shaped granules have also been observed in both samples. Most of the starch granules displayed a very smooth surface. Granules of Meirenhong sample ranged from 33.3 to 70.1 μm in length and 16.9 to 28.2 μm in width, while granules of Wawalian sample ranged from 33.9 to 92.7 μm in length and 14.6 to 29.3 μm in width. According to the ratio of long/short axis length, starch granules might be divided into three populations. Starch granule with the ratio of long/short-axis length below 1.1 was round in shape, starch granules with the ratio between 1.1 and 1.4 was oval shaped, and starch granules with the ratio above 1.4 was elongated in shape. In this study, the granule shapes of lotus rhizome starch mainly included two sorts: large elongated or oval-shaped granules and small round-shaped granules. The numbers of different shaped granules of the starches were shown in Table 4. In Meirenhong sample, the elongated and oval-shaped starch granules accounted for about 60% of the total granule number, and that was about 70% for Wawalian sample. The shapes of starches isolated from Meirenhong and Wawalian lotus rhizome were analogous with those of lotus root starch (Jane et al. 1994). The lotus root starch had oval- and round-shaped granules with long axis of 10–50 μm and short axis of 10–35 μm. The granule size in this study was also close to that of lotus rhizome starch granules determined by laser light-scattering analysis in Zhong's study (Zhong et al. 2007). Most of the tuber and root starches are simple granules, the exception being cassava and taro starches, which appear to be a mixture of simple and compound granules (Hoover 2001). The granule size is variable and ranges from 1 to 110 μm depending on the starch source (Hoover 2001). Most of the tuber and root starch granules are oval; however, round, spherical, polygonal, and irregularly-shaped granules are also found (Hoover 2001).

**Table 4:** Number of different shaped starch granules

Cultivar name	Number of different shaped starch granules		

	Elongated and oval-shaped granules	Round-shaped granules	Ratio
Meirenhong	125	71	1.76
Wawalian	137	60	2.28

### Gelatinization properties of starch

**Figure 6:**
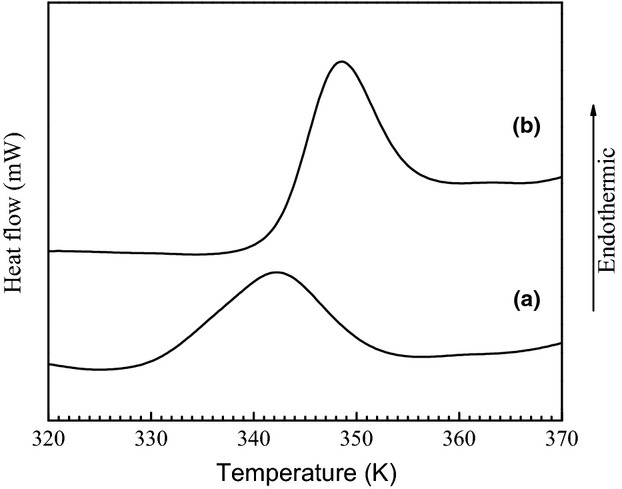
Differential scanning calorimetry (DSC) thermograms of starch sample (a) Meirenhong and (b) Wawalian.

The DSC thermograms of starch gelatinization were depicted in Figure 6 and the thermal parameters were given in Table 5. The gelatinization temperatures (*T*_o_, *T*_p_, and *T*_c_) of Meirenhong sample were lower than those of Wawalian sample. But the gelatinization range (*T*_c_–*T*_o_) for Meirenhong sample was 20.8 K, which was significantly wider than 13.0 K for Wawalian sample. The enthalpy values were 11.2 and 13.3 J g^−1^ for Meirenhong and Wawalian samples, respectively. The enthalpy of gelatinization values of starches has been reported to be affected by factors, such as granule shape and the relative degree of crystallinity.

**Table 5:** Thermal properties of lotus rhizome starches

Cultivar name	*T*_o_ (K)	*T*_p_ (K)	*T*_c_ (K)	*T*_r_ (K)	Δ*H* (J g^−1^)
Meirenhong	330.5	342.0	351.3	20.8	11.2
Wawalian	342.4	348.4	355.4	13.0	13.3

*T*_o_, onset temperature; *T*_p_, peak temperature; *T*_c_, conclusion temperature; *T*_r_, temperature range *T*_c_–*T*_o_; Δ*H*, enthalpy of gelatinization.

Bogracheva et al. (1998) studied the gelatinization of A- (maize), B- (potato), and C-type (pea) starches in excess water with, or without KCl (0.2–1.5 mol/L). Irrespective of the solute used, the A- and B-type starches showed narrow endothermic peaks. B-type starch gave a transition with lower peak temperature than A-type starch. The C-type starch showed a double peak curve in KCl solutions. The first peak of the double peak curve for C-type starch represented the melting of B polymorphs, and the second peak represented the melting of A polymorphs (Bogracheva et al. 1998). In this study, the gelatinization range for Meirenhong sample was significantly wider than that of Wawalian sample, which can be explained that the starches isolated from Meirenhong lotus rhizome was C-type crystalline containing both A- and B-type polymorphs, while Wawalian sample contained only A-type polymorphs.

### Pasting properties of starch

**Figure 7:**
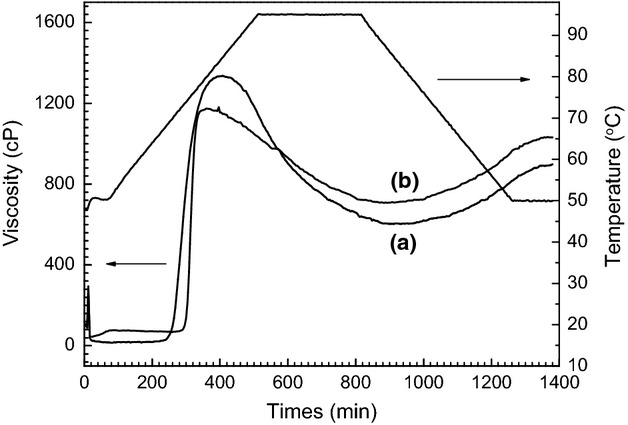
Rapid viscosity analyzer (RVA) pasting profiles at 6% starch concentration for (a) Meirenhong and (b) Wawalian.

There were obvious differences in RVA profiles of starches from Meirenhong and Wawalian samples (Fig. 7). The pasting parameters determined by RVA were summarized in Table 6. Viscosity of Meirenhong sample began to increase earlier than that of Wawalian sample, suggesting lower pasting temperature of Meirenhong sample. Wawalian sample showed higher hot and cool viscosities, and higher setback, but lower peak viscosity and breakdown than those of Meirenhong sample, which indicated that Wawalian sample had a relatively higher amylose content.

**Table 6:** Pasting properties of lotus rhizome starches

Cultivar name	Peak viscosity (cP)	Hot viscosity^1^ (cP)	Breakdown (cP)	Final viscosity (cP)	Setback (cP)	Peak time (min)	Pasting temp (K)
Meirenhong	1336	603	733	898	295	6.8	343
Wawalian	1181	707	474	1031	324	6.6	346

cP, centipoise.

1 Hot viscosity: the pasting viscosity after the holding time at 368 K.

RVA is considered to simulate food processing and is used to relate functionality to structural properties. In a typical RVA profile, the viscosity increases to a maximum, followed by a decrease to a minimum value as the granules rupture (referred to as the breakdown). As the temperature decreases, the viscosity again increases from the minimum to a final value, which is referred to as the setback (Copeland et al. 2009). According to the theory of Jenkins and Donald, water first enters the amorphous growth rings, and at a certain degree of swelling, disruptive stress is transmitted through connecting molecules from the amorphous to the crystalline regions (Jenkins and Donald 1998). Amylose molecules begin to leach from the granules as they are disrupted under shear and the viscosity of the resulting paste increases to a maximum, which corresponds to the point when the number of swollen but still intact starch granules is at a maximum. The maximum is followed by a decrease in paste viscosity, as the granules rupture and starch molecules are dispersed in the aqueous phase. As the starch paste cools, the viscosity increases due to the formation of a gel held together by intermolecular interactions involving amylose and amylopectin molecules. In gels that contain about 25% amylose, the starch molecules form a network resulting in a firm gel. On standing, starch gels retrograde and form insoluble B-type crystallites due to association of linear regions of *α*-(1→4) linked glucose units in the polymers (Jenkins and Donald 1998).

The RVA parameters have been correlated with texture and product quality (Copeland et al. 2009). The rate and extent of swelling and breakdown are dependent on the type and amount of starch, the temperature gradient, shear force, and the composition of the mixture, for example, the presence of lipids and proteins (Debet and Gidley 2007). In general, there is a negative relationship between the amylose content of starch and the gelatinization temperature and peak viscosity. The peak time and peak viscosity are indicative of the water-binding capacity of the starch and the ease with which the starch granules are disintegrated, whereas higher setback values are usually correlated with the amylose content of the starch (Copeland et al. 2009).

## Conclusions

In the studies described here, starches were isolated from rhizomes of two lotus cultivars, and their structure and physicochemical properties were studied. XRD and ^13^C CP-MAS NMR confirmed that starch in rhizome of cultivar Meirenhong exhibited C-type polymorph, while starch in rhizome of cultivar Wawalian showed A-type polymorph. The relative crystallinities of Meirenhong and Wawalian samples were quantitatively estimated to be 35% and 37% from XRD, and 39% and 41% from ^13^C CP-MAS NMR, respectively. ATR-FTIR results indicated that the external regions of the two samples had a great level of ordered structure. Granules of Meirenhong sample ranged from 33.3 to 70.1 μm in length and 16.9 to 28.2 μm in width, and the elongated and oval-shaped starch granules accounted for about 60% of the total granule number. Wawalian sample ranged from 33.9 to 92.7 μm in length and 14.6 to 29.3 μm in width, and about 70% were the elongated and oval-shaped starch granules. Gelatinization temperatures of starch in Meirenhong and Wawalian were 330.5 and 342.4 K, respectively. But the gelatinization temperature range for Meirenhong sample was 20.8 K, which was significantly wider than 13.0 K for Wawalian sample. Starch in Meirenhong showed lower pasting temperature, lower hot and cool viscosities, lower setback, and higher peak viscosity and breakdown than those of Wawalian in RVA pasting profiles at 6% starch concentration.
